# P-986. Primary barriers to implementing Antibiotic Stewardship in rural Georgia

**DOI:** 10.1093/ofid/ofaf695.1185

**Published:** 2026-01-11

**Authors:** Rayven Todd, Shondia Evans, Fantasia Gorham, Raybun Spelts, Damani Andre, Rafael Ponce-Terashima, Kenneth Onyedibe

**Affiliations:** Morehouse School of Medicine, Atlanta, Georgia; Georgia Hospital Association, atlanta, Georgia; Georgia Department of Public Health, atlanta, Georgia; Georgia State Department of Public Health, atlanta, Georgia; Morehouse School of Medicine, Atlanta, Georgia; Mercer University School of Medicine, Macon, Georgia; Morehouse School of Medicine, Atlanta, Georgia

## Abstract

**Background:**

Antibiotic Stewardship Programs (ASPs) are a critical component of improving public health considering the growing prevalence of antimicrobial resistant infections and a limited supply of antimicrobial options. Therefore, to identify barriers to implementing ASPs in rural Georgia, we administered surveys to participants of Antibiotic Stewardship Bootcamps in 2023 and 2024.

**Methods:**

The conferences featured speakers in infectious disease and public health who conducted didactic lectures, interactive sessions, and case-based learning in antibiotic stewardship. We administered post session online surveys to the participants (nurses, pharmacists, nurse practitioners) who attended the 2023 and 2024 sessions. Survey responses were evaluated using descriptive statistics.

**Results:**

There were 25 participants in 2023 and 40 in 2024. In both years, ≥ 95% of attendees completed the survey. Participants were from 14 hospitals in 2023 and 24 hospitals in 2024. More than 95% of participants agreed that the sessions improved their knowledge, competence, leadership skills, and practices in both 2023 and 2024. Responses from both years also showed that participants planned to implement changes in their work practice such as applying the latest best practice (≥ 75%), implementing multidisciplinary approaches (≥ 50%), and modifying approaches for adherence/compliance challenges (≥ 44%) following the conference (table 1). 100% of participants intended to change their current practice behavior and felt confident in their ability to implement these changes. However, at least 48% and 27.5% of participants stated that “Time constraint” and “Technology limitations”, respectively were perceived as important primary barriers of implementation (table 1). None of the participants believed that insurance or financial issues would be a primary barrier.

**Conclusion:**

Our findings show that time constraints as well as technology and digital limitations are the leading barriers to implementing ASP in rural Georgia and not financial or insurance concerns. Addressing the barriers to ASP in rural Georgia requires a multifaceted approach that includes technological and staffing improvements to overcome some of these barriers.

**Disclosures:**

All Authors: No reported disclosures

